# Success and failure of controlling the real‐time functional magnetic resonance imaging neurofeedback signal are reflected in the striatum

**DOI:** 10.1002/brb3.1240

**Published:** 2019-02-20

**Authors:** Leon Skottnik, Bettina Sorger, Tabea Kamp, David Linden, Rainer Goebel

**Affiliations:** ^1^ Department of Psychiatry and Neuropsychology Maastricht University Maastricht Netherlands; ^2^ Department of Cognitive Neuroscience Maastricht University Maastricht Netherlands; ^3^ Brain Innovation BV Maastricht Netherlands; ^4^ MRC Centre for Neuropsychiatric Genetics and Genomics, School of Medicine Cardiff University Cardiff United Kingdom; ^5^ School of Mental Health and Neuroscience Maastricht University Maastricht Netherlands; ^6^ Department of Neuroimaging and Neuromodeling Netherlands Institute for Neuroscience, an institute of the Royal Netherlands Academy of Arts and Sciences (KNAW) Amsterdam Netherlands

**Keywords:** neurofeedback, real‐time functional magnetic resonance imaging, self‐regulation, striatum

## Abstract

**Introduction:**

Over the last decades, neurofeedback has been applied in variety of research contexts and therapeutic interventions. Despite this extensive use, its neural mechanisms are still under debate. Several scientific advances have suggested that different networks become jointly active during neurofeedback, including regions generally involved in self‐regulation, regions related to the specific mental task driving the neurofeedback and regions generally involved in feedback learning (Sitaram et al., 2017, *Nature Reviews Neuroscience*, 18, 86).

**Methods:**

To investigate the neural mechanisms specific to neurofeedback but independent from general effects of self‐regulation, we compared brain activation as measured with functional magnetic resonance imaging (fMRI) across different mental tasks involving gradual self‐regulation with and without providing neurofeedback. Ten participants freely chose one self‐regulation task and underwent two training sessions during fMRI scanning, one with and one without receiving neurofeedback. During neurofeedback sessions, feedback signals were provided in real‐time based on activity in task‐related, individually defined target regions. In both sessions, participants aimed at reaching and holding low, medium, or high brain‐activation levels in the target region.

**Results:**

During gradual self‐regulation with neurofeedback, a network of cortical control regions as well as regions implicated in reward and feedback processing were activated. Self‐regulation with feedback was accompanied by stronger activation within the striatum across different mental tasks. Additional time‐resolved single‐trial analysis revealed that neurofeedback performance was positively correlated with a delayed brain response in the striatum that reflected the accuracy of self‐regulation.

**Conclusion:**

Overall, these findings support that neurofeedback contributes to self‐regulation through task‐general regions involved in feedback and reward processing.

## 1 INTRODUCTION

Despite its extensive use over several decades and broad evidence for neurofeedback induced changes that extend beyond the neurofeedback training environment (including on memory Young et al., 2017, affect Scheinost et al., 2013; Zilverstand, Sorger, Sarkheil, & Goebel, 2015, attention Zilverstand et al., 2017, perception Amano, Shibata, Kawato, Sasaki, & Watanabe, 2016 and motor performance Subramanian et al., 2011) the neural mechanisms underlying neurofeedback are subject of an ongoing debate (for an overview see Sitaram et al., 2017). In a recent meta‐analysis, whole‐brain activation during real‐time functional magnetic resonance imaging (rtfMRI) neurofeedback was compared across different neurofeedback studies (Emmert et al., 2016). Activation during neurofeedback training was observed in areas implicated in self‐regulation and cognitive control, as well as in areas recruited during visual feedback learning, even if these areas were not actually the target of the self‐regulation training. The activated network encompassed the dorsolateral (DLPFC) and ventrolateral prefrontal cortex (VLPFC), the temporo‐parietal cortex and the thalamus, anterior insula (aINS), the posterior section of the anterior cingulate cortex (pACC), visual areas and the basal ganglia, with several local maxima distributed over the striatum. Activity in these regions most likely reflects several different processes, including the preparation and execution of mental strategies supporting self‐regulation of brain activity, reward processing, self‐evaluation of performance based on feedback information and the updating of strategies, but an extensive body of research is still needed to disentangle these processes. To discriminate the neural basis of neurofeedback from networks also recruited during other forms of self‐regulation training, it remains to be understood which regions shared between different neurofeedback tasks are specific to neurofeedback and which are reflective of self‐regulation per se.

Marchesotti et al. (2017) detected a selective activation increase in the striatum during motor imagery with neurofeedback when comparing meta‐analytic activation maps of motor imagery with and without providing neurofeedback and Johnston, Boehm, Healy, Goebel, and Linden (2010) had reported increased activation in the ventral striatum with progression in neurofeedback training for up‐regulating negative affect by providing neurofeedback from individual areas that showed increased activation in response to negative affective image. In congruence with these reported activation increases in the striatum during neurofeedback, several theoretical frameworks note that BCI control/neurofeedback rewards subjects for a certain mental operation or neural state, notably by underling the crucial involvement of operant/instrumental conditioning in neurofeedback (Fetz, 2007), by interpreting BCI control training as skill learning that is heavily dependent on plasticity in the basal ganglia (Birbaumer, Ruiz, & Sitaram, 2013) or by underlining the importance of feedback loops for biofeedback learning in general (Lacroix & Gowen, 1981). While early EEG‐neurofeedback studies lacked direct evidence for involvement of the striatum in neurofeedback due to the limitations of EEG in coverage of subcortical areas (Grech et al., 2008), contemporary approaches on EEG and fMRI neurofeedback agree with regard to the central role of striatal reward learning (Birbaumer et al., 2013; Davelaar, 2018).

In the present study, we extended the aforementioned line of research by comparing self‐regulation with and without neurofeedback with a special focus on the striatum, a key region involved in feedback and reward processing (Balleine, Delgado, & Hikosaka, 2007; Bartra, McGuire, & Kable, 2013; Kohrs, Angenstein, Scheich, & Brechmann, 2012), the central hub of dopamine based reinforcement learning (Robbins & Everitt, 1996) where feedback information is processed and further utilized to guide actions (O'Doherty et al., 2004; Samejima, Ueda, Doya, & Kimura, 2005) and constituting the main hub for long‐term motivation of behaviour based on reward learning (Tricomi, Balleine, & O'Doherty, 2009), making the understanding of how neurofeedback affects the striatum a crucial element of understanding the facilitating effects of neurofeedback in general.

As neurofeedback is most commonly used to guide a participant's self‐regulation by reinforcing activation states via operant conditioning with positive feedback (Birbaumer et al., 2013; Fetz, 2007), we therefore predicted that striatum activation would constitute a crucial marker for differentiating between self‐regulation with neurofeedback and self‐regulation without neurofeedback, as it reflects external reward information that is utilized to guide ongoing behaviour (Balleine et al., 2007), which is lacking in self‐regulation without neurofeedback.

While the striatum is a functionally heterogeneous structure (parcellation studies suggest that ventral/anterior portions are more strongly involved in evaluating incoming reward, whereas medial to dorsal sections rather bias actions based on previously processed rewards (Balleine et al., 2007; Jung et al., 2014; O'Doherty et al., 2004)), different functional processes in the striatum transition smoothly into each other (Haber, Fudge, & McFarland, 2000) and timing of incoming rewards constitutes a crucial influence across different processing stages in the striatum (Cardinal, 2006; Gustavo, Soares, & Paton, 2015; McClure, Berns, & Montague, 2003; Pagnoni, Zink, Montague, & Berns, 2002). So while previous studies have mainly concentrated on localizing the regions involved in neurofeedback, we additionally applied a time‐resolved analysis on the blood oxygen level dependent (BOLD) signal of the striatum, to determine the temporal properties of feedback processing.

Up to this date, most neurofeedback paradigms focused on decreasing or increasing activation within a certain brain region (Caria et al., 2007; Hamilton, Glover, Hsu, Johnson, & Gotlib, 2011), functional connectivity between brain regions (Megumi, Yamashita, Kawato, & Imamizu, 2015), directional connectivity between brain regions (Haller et al., 2013), or frequency‐bands (Gevensleben et al., 2009; Mottaz et al., 2015). These paradigms reinforced subjects to modulate the neurofeedback signal into one direction, that is, to either up‐ or down‐regulate the neurofeedback signal maximally. We recently demonstrated feasibility of a novel type of neurofeedback paradigm in which participants focused on achieving and maintaining a specific target *level *of activation (Sorger, Kamp, Weiskopf, Peters, & Goebel, 2018). Participants aimed at reaching/maintaining a rtfMRI‐neurofeedback signal (visualized by means of a thermometer display) corresponding to the brain‐activation level within individually defined brain regions at either 30%, 60% or 90% of their individual maximal activation capacity. We found that participants showed a significantly increased ability to gradually self‐regulate activation in the neurofeedback target regions, when receiving visually presented neurofeedback information compared to gradual self‐regulation without providing neurofeedback. In contrast to classical paradigms that train to maximize (de)activation or connectivity, participants trained according to the novel parametric activation paradigm received detailed neurofeedback information on the current brain‐activation level with every data point (here every 2 s) visualized as deviation of the actual condition (actually achieved brain‐activation level) from the different nominal conditions (instructed target brain‐activation levels). Moreover, they could deviate from the task goal by both reaching too high or too low activation levels (not given in the conventional, maximization paradigms).These features considerably increase the general task difficulty, and we would expect that successful task performance is being experienced as strongly rewarding (see DePasque Swanson & Tricomi, 2014). Another advantage of gradual feedback for studies into the mechanisms of self‐regulation is that the visual information provided during neurofeedback is more important for successful task performance than in maximization paradigms, as participants not only need to learn how activation could be increased or decreased best, but also how the actual magnitude of activation can be held at a particular target level. Gradual feedback protocols are thus particularly suited for studies that look into the learning mechanisms underlying successful neurofeedback training.

In the present study, we defined and applied a novel marker of self‐regulation success to a dataset from the aforementioned self‐regulation study by Sorger et al. (2018). This marker of self‐regulation success represents the neurofeedback reward value as indicated by the visual information on the feedback display. In the study of Sorger et al. (2018), each participant chose one individual mental task for self‐regulation and all participants trained to self‐regulate their engagement with the chosen mental content gradually (chosen mental tasks included inner speech, motor imagery, mental calculation, visual imagery and auditory imagery). The inter‐individual heterogeneity of self‐regulation strategies allows investigating the shared neural basis of neurofeedback. Participants underwent two self‐regulation sessions during fMRI, one with and one without receiving feedback information from individually defined neurofeedback target regions. This allows us to control for effects of self‐regulation that are unrelated to neurofeedback as for example observed during meditation (Kjaer et al., 2002; Tang, Hölzel, & Posner, 2015), and reveal regions more related to the actual processing of neurofeedback and the implicated reward information. As the neurofeedback signal was provided continuously, the constant influx of feedback information created a demanding situation for the processing of reward information. Neurofeedback was constantly updated, while being delayed over several seconds in relation to the mental action actually causing a change in the neurofeedback signal. As activation in the striatum is known to be strongly influenced by the temporal properties of reward information (Cardinal, 2006; Gustavo et al., 2015; McClure et al., 2003; Pagnoni et al., 2002), analysis has to take the temporal sensitivity of reward processing into account. Analysis of the available data therefore focused on the dynamic and delayed nature of the reward information provided by rtfMRI neurofeedback. This was achieved by extracting one value of neurofeedback performance for every data point acquired during gradual self‐regulation periods (every 2 s) and by relating this information to striatum activation in different time windows.

Taking into consideration these ideas and in order to further study the neuronal mechanisms of rtfMRI neurofeedback, more particularly the role of the striatum, the present study focused on the following research objectives:

- Demonstrate joint activation of cortical control areas and areas related to feedback learning *within a single sample* during neurofeedback‐guided self‐regulation compared to rest, thereby investigating the replicability of recent meta‐analytical findings (combining data of several neurofeedback studies Emmert et al., 2016) and their reliability in smaller samples.
- Separate activation related to feedback processing from activation related to self‐regulation during neurofeedback in the striatum and determine whether increased striatum activation during neurofeedback reflects a specific response to the information contained in the provided neurofeedback information.
- Disentangle which activation increases during neurofeedback indicate feedback processing and which are reflective of higher‐order cognitive control processes involved in self‐regulation.

## 2 METHODS AND DESIGN

### 2.1 Participants

All analyses were performed on the dataset acquired by Sorger et al. (2018): 10 healthy participants (mean age: 27.0 years, *SD*: 3.8 years, five female, one left‐handed), all students or staff members of the Faculty of Psychology and Neuroscience at Maastricht University with normal or corrected‐to‐normal vision participated in the study (see Sorger et al., 2018 for more detailed participant characteristics). None of the participants had participated in a neurofeedback experiment before. Before each MRI scanning session, participants gave written informed consent. The experimental procedure was approved by the local Ethics Committee of the Faculty of Psychology and Neuroscience at Maastricht University.

### 2.2 Experimental design

Preceding the first MRI measurement, each participant freely chose one individual mental task for self‐regulation: Experimenters suggested various mental tasks (inner speech, motor imagery, mental calculation, visual imagery and auditory imagery) that had been proven to evoke robust brain activation in circumscribed brain regions in previous fMRI studies as possible activation strategies. Additionally, the experimenters recommended several modulation strategies that could be applied by participants to alter the brain‐activation level. Basically, these strategies allowed for changing certain aspects of mental‐task performance parametrically (e.g., the speed, intensity or complexity). Participants selected their activation strategies and initial modulation strategies based on personal preference or feeling of best mastery. Chosen self‐regulation tasks included inner speech, motor imagery, auditory imagery, and visual imagery. Importantly, no participant used voluntary emotion regulation as mental strategy, thereby forecoming that alterations in striatum activation were dominated by voluntarily generated affective states.

Participants received no feedback in one fMRI session, whereas in the other session they were provided with real‐time information on the current BOLD‐signal level in a predefined mental task‐related brain region. During neurofeedback sessions, participants were asked to modulate their BOLD signal to three different target levels using the chosen mental task. The no‐feedback and feedback fMRI sessions took place on separate days for all participants. The order of the type‐of‐training conditions (no feedback‐feedback or feedback‐no feedback) was balanced across participants. Both scanning sessions consisted of four training (modulation) runs in which participants were visually instructed to modulate their BOLD‐signal magnitude to the three different target levels. Each target‐level condition appeared three times per run in randomized order resulting in a total of twelve trials per target‐level and type‐of‐training condition. Each of the nine modulation blocks and each of the ten resting blocks that alternated with the modulation blocks lasted 26 s resulting in a run length of 8 min and 14 s. A feedback scanning session started with a functional‐localizer run in order to select a mental task‐specific neurofeedback target region and to determine the individual maximum percent signal change (maxPSC). In the functional‐localizer run, two target‐levels (50% and 100%) were implemented (five trials per target‐level condition). The two target‐level conditions appeared in alternating order. Again, the duration of the 10 modulation trials and the eleven resting periods were 26 s adding up to a total run duration of 9 min and 6 s.

### 2.3 Task instructions

Participants were instructed to keep their selected activation strategy constant across all functional runs (functional‐localizer, no‐feedback and feedback runs). Thus, they should not change their general activation strategy across time (and sessions). In order to modulate their BOLD signal to the different target levels, participants were asked to apply the modulation strategies. Importantly, in the feedback condition participants were instructed to consider the provided neurofeedback information and to explore which of the modulation strategies were most effective. Moreover, participants were explicitly allowed to adapt the suggested modulation strategies or even generate and test novel (“own”) modulation strategies. During functional‐localizer and no‐feedback runs, participants were asked to try to evoke different brain‐activation levels based on their current hypothesis on how the BOLD‐signal magnitude can be altered systematically.

**Figure 1:**
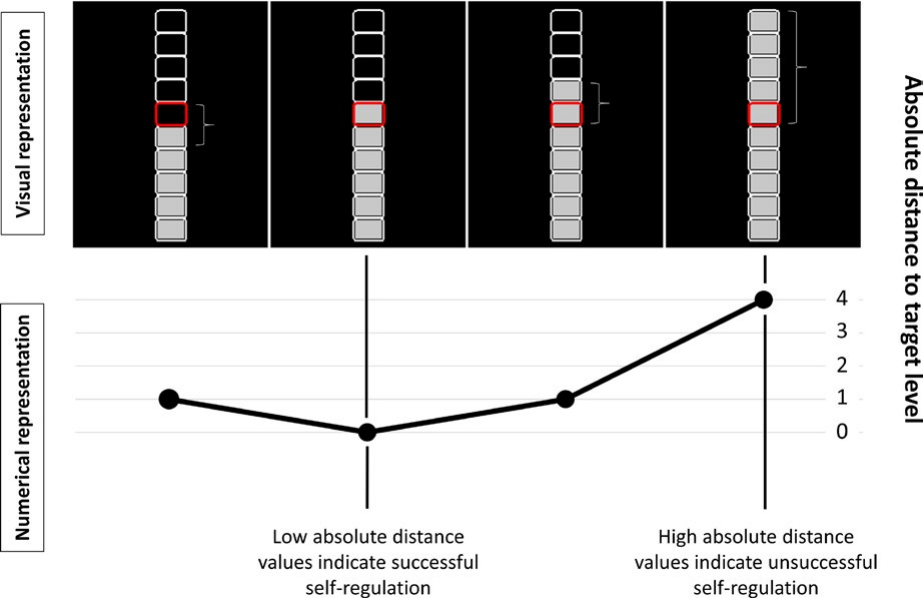
Absolute distance of achieved activation level to instructed target activation level. Participants evaluated the appropriateness of their mental operation (and therewith their self‐regulation success) based on the visually provided neurofeedback information. They could assess their self‐regulation success by obtaining the absolute distance between the magnitude of the actually achieved activation level (provided neurofeedback information) and the instructed target activation level (indicated by the red rectangular). A smaller and larger distance to the target activation level represented a superior and inferior self‐regulation performance, respectively

During self‐regulation (with and without feedback), a thermometer‐like display on black background was used consisting of 10 white rectangles stacked on top of each other (see Figure 1). Participants were instructed to adjust their BOLD‐signal magnitude to a particular target level by displaying the outline of a certain rectangle in red for the duration of the modulation trial. Thus, the vertical position of the colored rectangle represented the desired brain‐activation target level. In the functional‐localizer run, rectangle 5 (counted from bottom) corresponded to the 50% condition and rectangle 10 represented the 100% condition of the individual maxPSC. In the modulation runs, rectangles 3, 6, and 9, corresponded to the low, medium, and high target‐level conditions, respectively. During resting periods, no rectangle was colored red. In the modulation runs of the feedback session, participants were additionally provided with continuously updated information about their current BOLD‐signal level within the neurofeedback target region. This was realized by filling in (with gray color) the thermometer's rectangles according to the actual current BOLD‐signal level within the neurofeedback target region. Participants were instructed to reach and hold the desired brain‐activation target level, thereby reducing the absolute distance between the BOLD‐signal level and the target rectangle (see Figure 1).

### 2.4 Data acquisition

(f)MRI data were acquired using a 1.5‐T whole‐body (Magnetom Sonata; Siemens AG, Erlangen, Germany) or a 3‐T head scanner (Siemens Allegra, Siemens AG). Participants' heads were fixated with foam padding to minimize spontaneous or task‐related motion. The proportion of participants undergoing 1.5 and 3T scanning was balanced (5/5) and each participant underwent the same field strength for both training sessions.

#### 2.4.1 Structural data acquisition

All participants received a high‐resolution T1‐weighted anatomical scan using a three‐dimensional (3D) magnetization prepared rapid‐acquisition gradient‐echo sequence (1.5‐T scanning: 192 slices, slice thickness = 1 mm, no gap, repetition time [TR] = 2000 ms, echo time [TE] = 3.93 ms, flip angle [FA] = 15, field of view [FOV] = 250 × 250 mm^2^, matrix size = 256 × 256, total scan time = 8 min and 34 s; 3‐T scanning: 192 slices, slice thickness = 1 mm, no gap, TR = 2,250 ms, TE = 2.6 ms, FA = 9, FOV =256 × 256 mm^2^, matrix size = 256 × 256, total scan time = 8 min and 26 s).

#### 2.4.2 Functional data acquisition

Repeated single‐shot echo‐planar imaging was performed. Except for the number of acquisitions (functional‐localizer run: 273 volumes; modulation runs: 247 volumes), identical scanning parameters were used for all functional measurements (TR = 2000 ms, TE = 40 ms, FA = 90, FOV = 224 × 224 mm^2^, matrix size = 64 × 64, number of slices = 25, slice thickness = 3 mm, 1 mm gap, slice order = ascending/interleaved).

## 3 DATA ANALYSIS

### 3.1 Selection and definition of neurofeedback target regions

After completion of the functional‐localizer run, the first two volumes were discarded from further analysis to account for T1‐saturation effects. Functional data were then preprocessed (motion correction, linear‐trend removal, temporal high‐pass filtering [three cycles/time course]). Eventually, a multiple‐regression general linear model (GLM) was calculated voxel‐wise applying predictors corresponding to the two target‐level conditions (predictor time courses being derived from a boxcar function convolved with a standard hemodynamic response function (single‐gamma function Boynton, Engel, Glover, & Heeger, 1996). Candidate neurofeedback target regions were identified by contrasting the mean brain activation during both target‐level conditions to the mean activation during the interleaved resting periods. From the obtained F‐maps (*p* < 0.05, Bonferroni‐corrected), a region of interest (ROI) was defined for each participant (for details of neurofeedback target regions see Sorger et al., 2018).

### 3.2 Calculation of the feedback signal

For an extensive description of how the neurofeedback signal was created the reader is referred to Sorger et al. (2018). In short, functional images were reconstructed and written to the scanner console's hard disk in real‐time during neurofeedback sessions. The real‐time data analysis software (Turbo‐BrainVoyager, Brain Innovation B.V., Maastricht, the Netherlands) was used to extract and average the BOLD‐signal values of all voxels in the individual neurofeedback target region at each TR. The resulting means were normalized to the range [0–10], in relation to the preceding baseline period (constituting a value of 0) and an individual maximum percent‐signal change value, derived from the localizer run (constituting a value of 10). The resulting value range was binned into 10 segments and all segments of the thermometer display up to the given feedback value were greyed. Feedback was updated every 2 s.

### 3.3 (f)MRI data preprocessing

To answer the specific research questions of the current paper, offline analysis of the (f)MRI data was performed using BrainVoyager QX (v2.8, Brain Innovation, Maastricht, the Netherlands). Anatomical data sets were corrected for spatial intensity inhomogeneity. For all participants, the anatomical data set from the first session was transferred into ACPC space and the anatomical data set from the second session was automatically aligned to the ACPC version of the first data set. Both data sets were spatially normalized by Talairach transformation. All functional data sets underwent slice scan‐time correction and temporal high‐pass filtering (three cycles per time course). Three‐dimensional (3D) head‐motion detection and correction was applied by spatially aligning all functional volumes of a session to the first functional volume of the first run within that session. Finally, all functional runs were spatially normalized to Talairach space and interpolated to a 3‐mm^3^ voxel resolution. For whole‐brain and masked analysis, functional data were smoothed in 3D with a 4‐mm FWHM Gaussian kernel.

### 3.4 Extraction of striatum time‐series

**Figure 2:**
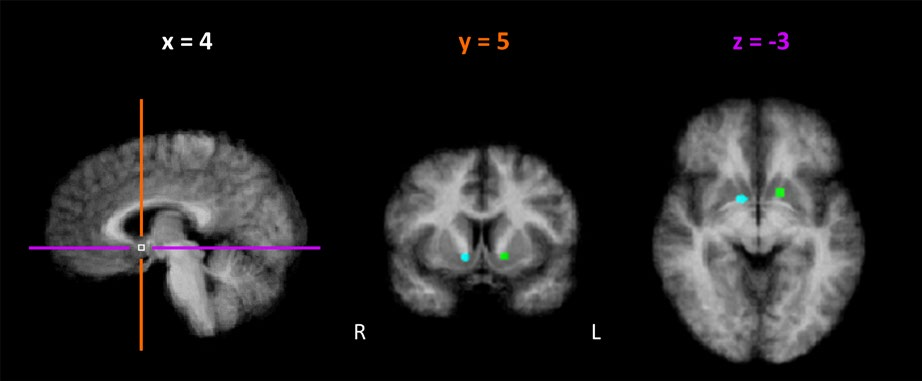
Definition of striatum regions of interest. The figure shows the right‐ and left‐hemispheric striatum regions of interest (R = right, L = left) overlaid on the mean of all individual anatomical data sets and slice positions of displayed coronal (orange) and axial (purple) slices. Regions of interests included all voxels in a 3‐mm sphere centered around peak coordinates from a recent meta‐analysis on reward processing representing maximal overlap of BOLD‐signal increase in response to positive reward (Bartra et al., 2013). Provided coordinates are in Talairach space

One ROIs for the striatum was defined for each hemisphere based on peak coordinates from a recent meta‐analysis on reward processing in fMRI (see Figure 2). For both hemispheres, selected coordinates marked the maximal spatial overlap of activation increases in response to reward of 126 fMRI studies (Bartra et al., 2013). The MNI coordinates reported in the meta‐analysis were converted into Talairach coordinates (Talairach & Tournoux, 1988) using the Yale BioImage Suite Package tal2mni tool (Lacadie, Fulbright, Rajeevan, Constable, & Papademetris, 2008). Spherical volumes of interest (one left‐ and one right‐hemispheric) with a 3‐mm radius were created around the particular coordinates. Both ROIs were located in the anterior section of the corpus striatum, centered between caudate head and anterior putamen (left striatum: 123 voxels, *x* = −14, *y* = 7, *z* = −2, right striatum: 123 voxels, *x* = −11, *y* = 3, *z* = −3; see Figure 2). The described approach increased the probability of detecting reward‐related activation in the brain while not making a priori assumptions regarding anatomical sub‐regions of the striatum contributing to the processing. For further analysis, the resulting striatum ROI in the hemisphere of the individual neurofeedback target region was chosen for each participant. As cortico‐striatal structural connectivity is known to be dominantly ipsilateral (see for example Innocenti, Dyrby, Andersen, Rouiller, & Caminiti, 2016, Jarbo & Verstynen, 2015) this approach increased the probability of detecting striatal activation specifically related to the cortical processes involved in the individual self‐regulation task. For each ROI, eight time courses (from each of the four self‐regulation runs per training condition) were extracted per participant.

Moment‐to‐moment neurofeedback stimulation images (as presented to the participant during the neurofeedback experiment) were re‐created applying the same procedure as described in Sorger et al. (2018) for the feedback sessions and the same procedure was applied post hoc for no‐feedback sessions, resulting in one picture per TR/scanned volume during modulation periods for both types of training. Using in‐house software written in MATLAB (v8.1 R13; The MathWorks, Natick), values of feedback magnitude were extracted from picture files. For each TR, one index of self‐regulation accuracy was created by calculating the absolute difference between the target level and the feedback magnitude actually achieved by the participant. Subsequently, the time series of accuracy indices were convolved with a hemodynamic response function to create a self‐regulation performance‐predictor time course (representing the neurofeedback task accuracy at each TR across time) for subsequent correlation analysis with ROI time‐courses.

### 3.5 Statistical analysis

#### 3.5.1 Whole‐brain analysis

To determine whether our sample showed activation during neurofeedback in coherence with recent meta‐analytical evidence on neurofeedback, we analyzed whole‐brain data in BrainVoyager QX by computing a group random‐effects GLM, including the types of training (feedback, no feedback), target levels (low: 30%, medium: 60%, high: 90% of the individual maxPSC), as well as six motion parameters as confounding predictors to estimate beta values. We employed a two‐way within‐subject design with *target‐level* (low, medium and high) and *type of training* (no feedback and feedback) as factors. Subsequently, we compared activity increases during self‐regulation with neurofeedback to passive viewing of neurofeedback (i.e. the resting condition) by contrasting activation across all target‐level conditions during modulation periods with neurofeedback to baseline, during which participants passively observed fluctuations in the neurofeedback signal.

#### 3.5.2 Striatum ROI analysis

To determine whether striatum activation increases during rtfMRI neurofeedback‐based self‐regulation compared to self‐regulation without neurofeedback and whether this effect is influenced by the height of the desired target level, we performed a standard volume of interest analysis in BrainVoyager QX: Time‐courses of all voxels within the meta‐analytically defined striatum ROIs were averaged to create one time‐course of each functional run. By computing a group random‐effects GLM on the striatum ROI time‐courses, including the HRF‐convoluted predictors for types of training (feedback, no feedback), target levels (low: 30%, medium: 60%, high: 90% of the individual maxPSC), as well as six motion parameters as confounding predictors to estimate beta values. We employed a two‐way within‐subject design with *target‐level* (low, medium, and high) and *type of training* (no feedback and feedback) as factors. A two‐way repeated measures analysis of variance (ANOVA, *F*‐test) with factors for *target level* and *type of training* was performed on the resulting striatum beta estimates.

#### 3.5.3 Time‐resolved analysis of neurofeedback performance and striatal activation

**Figure 3:**
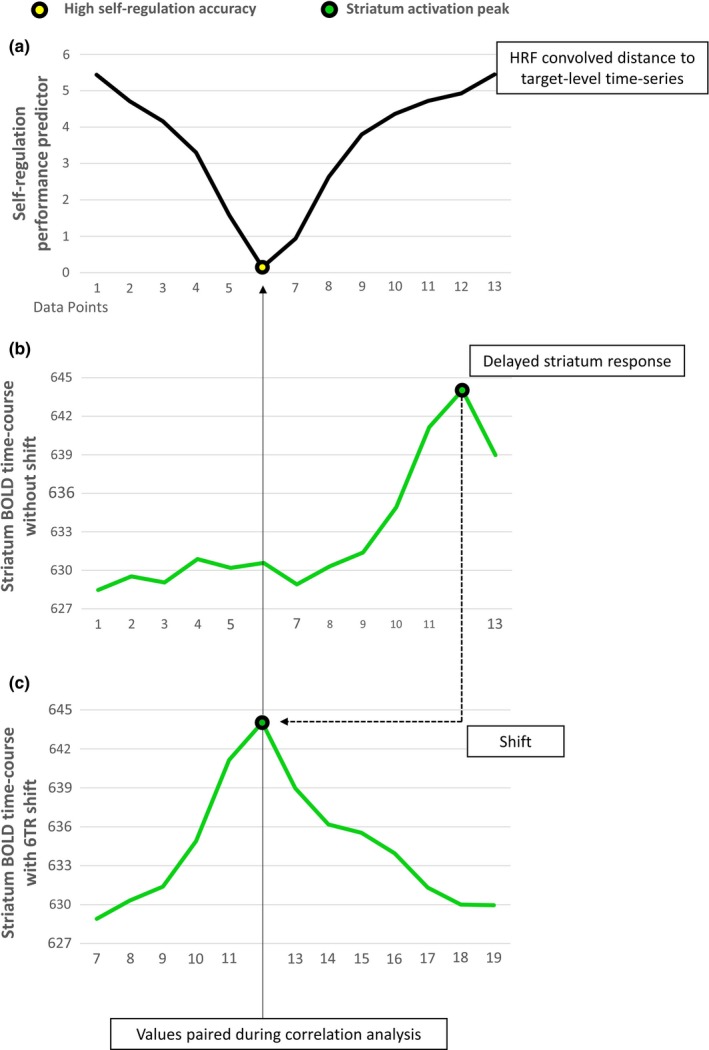
Time‐resolved analysis of striatum activation in response to self‐regulation success. The figure displays the logic of the performed correlation analysis. Simulated data during gradual self‐regulation is shown: (a) An HRF‐convolved time series of performance indices is created from the absolute distance to the target activation level. Successful self‐regulation (i.e., accurate regulation of the feedback signal to the target activation level) is represented by a low value. (b) When a corresponding activation increase in the striatum ROI is delayed (in this example 6 TR), the activation peak is not paired to the improvement in performance during correlation analysis. (c) Only, when the striatum time‐course is shifted 6 TRs backwards, the increase in striatum activation is aligned to the decrease in absolute distance during correlation analysis

To investigate whether striatum activation during neurofeedback is modulated by the displayed information on self‐regulation accuracy, performance‐predictor time courses were correlated to striatum time courses. In order to also detect temporally delayed activation changes, the predictor time courses were shifted in time (see Figure 3). For all time points during the modulation periods, predictor time courses were correlated to the striatum‐ROI time courses within runs. One correlation coefficient was acquired separately for each temporal shift, with the maximum shift being seven TRs, resulting in eight correlation coefficients per run (including the correlation coefficient for the nonshifted time course).

Correlation coefficients were subsequently Fisher *z*‐transformed and first averaged within subjects and conditions. To create stable estimates of the correlation between the two variables with expected high variability, resulting correlation means were temporally smoothed by averaging the *z*‐transformed correlation means within two time‐windows that were sufficiently distant in time to capture different BOLD response peaks: The early time window (0–3 TR shifts) included immediate BOLD changes with a margin for variability in BOLD timing and shape and delay in neural reactions. The late time window (4–7 TR shifts) included BOLD changes delayed for at least 8 s after an immediate BOLD response would be expected, so that BOLD changes in the late time window rather reflect a secondary stage of processing, as for example response preparation, not an immediate reaction to the rewarding feedback.

This procedure resulted in one correlation coefficient per subject, time window and type of training (four correlation coefficients per participant). *Z*‐transformed correlation coefficients were compared between types of training separately within the two time windows using student's paired‐sample *t* tests and applying Bonferroni correction for multiple comparison correction. Effect sizes were calculated based on Cohens D (Cohen, 1988) adapted for paired measures (Morris & DeShon, 2002).

#### 3.5.4 Masked voxel‐wise analysis of the neurofeedback network

To identify activation increases during neurofeedback independent of self‐regulation across the whole neurofeedback network, the voxel‐wise group random‐effects GLM was restricted to a mask constituting of voxels within 15‐mm radii around cortical and 20‐mm radii around the two subcortical meta‐analytic peak voxels, that marked activation increases across several neurofeedback studies compared to rest, using the peak voxels described by Emmert et al. (2016) in the pACC, aINS, vlPFC, dlPFC, temporo‐parietal and occipital cortex, and two subcortical peak coordinates that constituted local maxima of several subcortical substructures (putamen, caudate, nucleus accumbens, globus pallidus, thalamus). The coordinates were transferred into Talairach space using the Yale BioImage Suite Package tal2mni tool (Lacadie et al., 2008). Differences between the two training conditions were compared by contrasting activation during modulation periods with neurofeedback to modulation periods without neurofeedback across the three target levels. Results were cluster corrected using Monte‐Carlo simulations with 1,000 iterations, a FWHM of 1,608 with an initial threshold of *p* < 0.01. Additionally, a liberal correction threshold was applied deliberately for decreasing the likelihood of missing potentially lower/more scattered activation in prefrontal control areas.

## 4 RESULTS

### 4.1 Effect of self‐regulation

**Figure 4:**
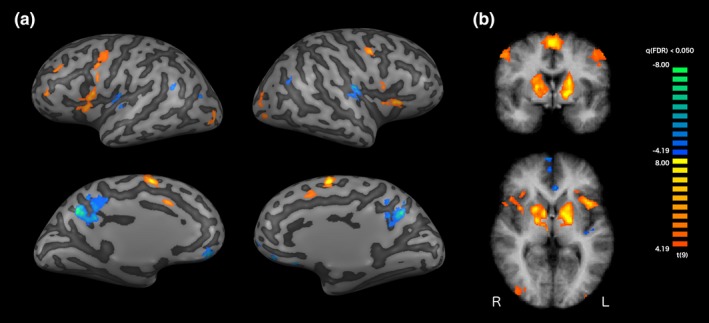
Self‐regulation with neurofeedback compared to passive viewing of neurofeedback. (a) In comparison to the rest condition, self‐regulation with neurofeedback was accompanied by increased activation in prefrontal control regions and regions involved in feedback processing (visual cortices, anterior insula) as well as decreased activation in the default mode network and the posterior insula. (b) An extended increase in subcortical activation was present during self‐regulation with neurofeedback, encompassing the striatum, thalamus, claustrum and the brainstem. The figure shows the whole‐brain RFX contrast map thresholded at FDR corrected *q* < 0.05 on a sample participant's inflated cortex segmentation (a) and on the average of the individual anatomical data sets (b)

The contrast for self‐regulation with neurofeedback compared to passive viewing of neurofeedback (i.e. rest) revealed an extensive network of regional increases (FDR corrected, *q* < 0.05), encompassing the bilateral precentral gyrus, the bilateral aINS, bilateral visual cortices, bilateral dorsolateral prefrontal cortex (dlPFC), left VLPFC, bilateral supplementary motor area, bilateral posterior pACC, left frontopolar cortex and an extensive subcortical cluster encompassing the striatum, thalamus and claustrum and deactivation (Figure 4) across the bilateral default mode network (transverse temporal gyrus, angular gyrus, precuneus, medial prefrontal cortex [mPFC]) and the posterior insula (pINS) bilaterally (Table 1). No significant differences between target levels or interactions were observed.

**Table 1:** Self‐regulation with neurofeedback compared to passive viewing of neurofeedback

Increased activation	Peak voxel coordinates			Peak voxel statistics	
Visual feedback and reward	*x*	*y*	*z*	*t*	*p*
Subcortical L/anterior insula L	−18	−4	4	12.3283	0.000001
Anterior insula R	33	20	10	10.0164	0.000004
Subcortical R	18	8	16	9.4796	0.000006
Visual cortex L	−39	−82	−9	6.2985	0.000141
Visual cortex R	36	−85	7	5.8471	0.000245
*Self‐regulation and attention*					
Supplementary motor area bilateral	3	−1	58	13.0617	0.000001
Ventrolateral PFC/precentral gyrus L	−60	11	16	8.7361	0.000011
Dorsal anterior cingulate bilateral	0	14	43	7.7295	0.000029
Precentral gyrus L	−39	29	28	7.2432	0.000049
Precentral Gyrus R	51	2	46	7.2152	0.000055
Frontopolar/dorsolateral PFC L	−45	48	13	7.0833	0.000058
*Decreased activation*					
Precuneus	−3	−55	31	−10.6369	0.000002
Posterior insula R	39	−22	19	−9.4743	0.000006
Posterior insula L	−51	−22	13	−8.4177	0.000015
Medial PFC	0	41	−9	−7.5212	0.000036
Posterior temoral cortex/angular gyrus L	−60	−58	23	−6.7828	0.000081
Posterior temoral cortex/angular gyrus R	48	−61	25	−6.7624	0.000082

Note

Self‐regulation with neurofeedback was accompanied by increased activation in prefrontal control regions (dorsolateral and ventrolateral PFC, dorsal anterior cingulate, precentral gyrus, supplementary motor area) and regions involved in feedback processing (visual cortices, anterior insula and an extended subcortical cluster) as well as decreased activation in the default mode network and the posterior insula. The table contains coordinates and statistics of peak voxels for the whole‐brain RFX contrast map thresholded at FDR corrected *q* < 0.05 (coordinates in Talairach space). PFC, Prefrontal cortex; L, left; R, right.

### 4.2 Effect of neurofeedback information on striatum activation

**Figure 5:**
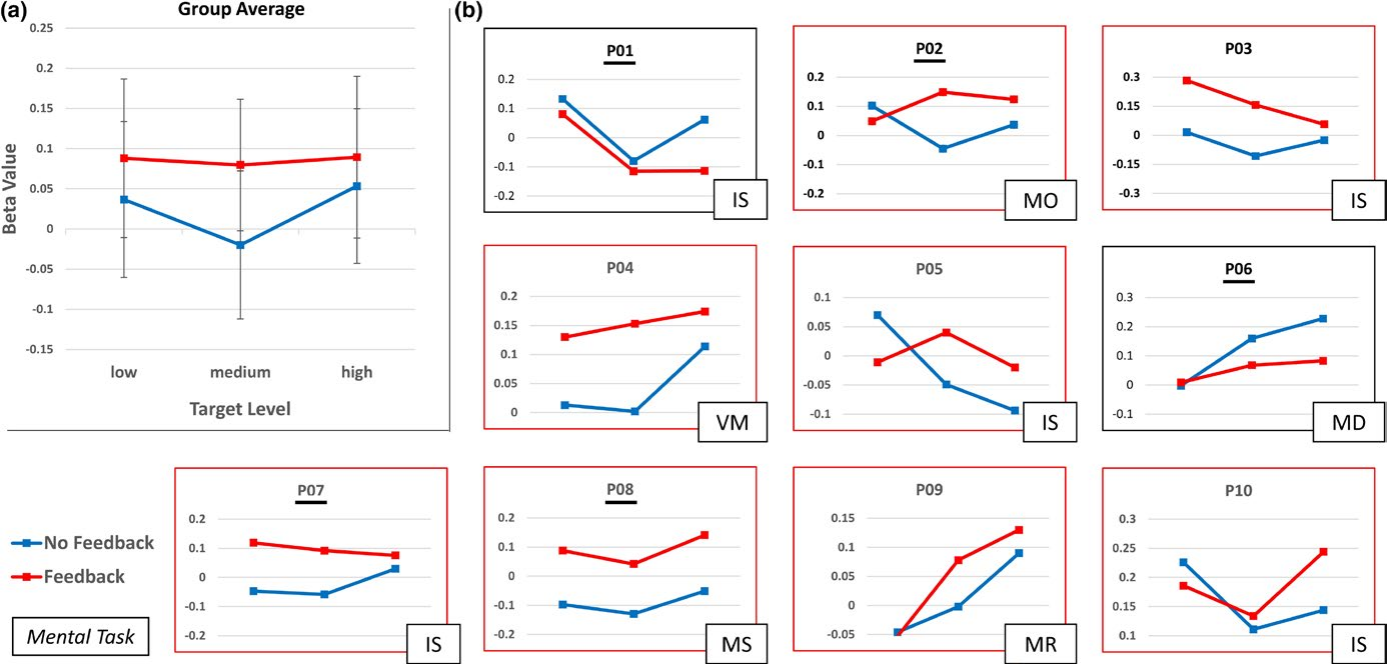
Effect of gradual self‐regulation success on striatum activation (group and single‐subject results). The figure visualizes the BOLD‐signal level within the striatum region of interest ipsilateral to the neurofeedback target region for the two type‐of‐training conditions and across the different target‐level conditions: (a) Mean beta values for each target‐level condition across all participants separately for the no‐feedback (blue) and feedback (red) condition. Error bars represent standard errors of the means. When pooling the data across the target‐level conditions, the difference of mean‐beta values for the two type‐of training conditions (feedback, no‐feedback) was significant (*p* < 0.05, Bonferroni‐corrected, one‐sided). (b) Single‐subject mean beta values separately for each target‐level and type‐of‐training condition. In 80% of participants (red‐rimmed), the mean striatum activation (i.e., pooled activation across the three target‐level conditions) was higher in the feedback compared to the no‐feedback condition. *Remark *.Participants with black underline underwent the feedback condition first and no‐feedback condition second. Abbreviations for mental strategies: IS = Inner speech, MO = mental orchestra, VM = visual motion imagery, MD = mental drawing, MS = mental sounds, MR = mental running

The main effect for *type of training* was significant (*p* = 0.036, one‐sided, Figure 5a) but there was no main effect of *target level *(*p* = 0.14, one‐sided) and no significant interaction (*p* = 0.08, one‐sided). Correspondingly, eight out of ten participants showed increased mean beta values during self‐regulation *with* neurofeedback compared to self‐regulation *without* neurofeedback but striatum activation did not differ in a consistent fashion between target levels across participants (see Figure 5b).

### 4.3 Modulation of striatum activation by self‐regulation success

An extensive analysis of task performance in the given sample can be found in Sorger et al. (2018). In short, participants were able to increase the BOLD signal magnitude to target levels in a gradual fashion across both training conditions (no‐feedback and feedback), but most participants demonstrated slightly increased ability to differentiate between target levels when provided with neurofeedback information. Both training conditions were matched closely with regard to the absolute distance to the desired target level (absolute distance mean (feedback) = 3.866, *SEM* = 0.19; mean (no feedback) = 3.858, *SEM* = 0.22; *p* = 0.96).

**Figure 6:**
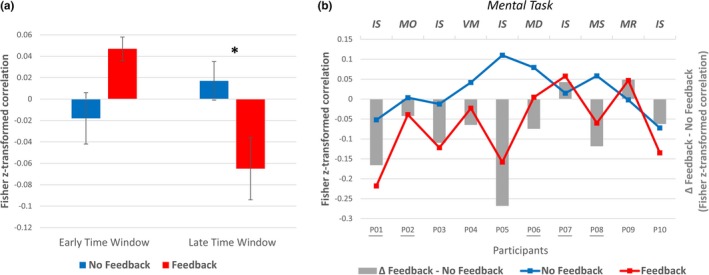
Relationship between self‐regulation success and striatum activation level (group and single subject results). Relationship between absolute distance to target activation level and striatum activation separately for the two type‐of‐training conditions. (a) Mean Fisher *z*‐transformed correlation coefficients between self‐regulation success and striatum activation separately for an early time window (0–3 TR shift, immediate and slightly delayed striatum activation) and a late time window (4–7 TR shift, delayed striatum activation). The difference of the correlation values with respect to the two type‐of‐training conditions (feedback, no feedback) was only significant for the late time window (*p* < 0.05, Bonferroni‐corrected, one‐sided). (b) Single‐subject results for the late time window. Eighty percent of participants showed a more negative correlation between distance to target‐level and striatum activation during gradual self‐regulation when receiving neurofeedback. *Remark*. Participants with black underline underwent feedback condition first and no‐feedback condition seconds. Abbreviations for mental tasks: IS = Inner speech, MO = mental orchestra, VM = visual motion imagery, MD = mental drawing, MS = mental sounds, MR = mental running

Correlation analysis between performance‐predictor time courses and the striatum time courses resulted in one mean *z*‐transformed correlation coefficients per type‐of‐training condition (feedback, no feedback) and the two predefined time windows (early, late): Early time window: Neurofeedback‐*r_z_* = 0.047, *SEM* = 0.01; No‐feedback‐*r_z_* = −0.018, *SEM* = 0.02; Late time window: Neurofeedback‐*r_z_* = −0.065, *SEM* = 0.03; No‐feedback mean *r_z_* = 0.017, *SEM* = 0.02 (Figure 6a). Subsequent paired *t* tests between training types indicated a significant difference between correlation coefficients only for the late time window (*p* = 0.044 (Bonferroni corrected), Cohens *d* = 0.912) but not for the early time window (*p* = 0.13 (Bonferroni corrected), Cohens *d* = −0.677). The effect was consistent across participants (see Figure 6b): 8 out of 10 participants showed a more negative mean correlation during gradual self‐regulation with feedback compared to gradual self‐regulation without feedback in this time window.

### 4.4 Sub‐components of the neurofeedback network involved in feedback processing

Voxel‐wise analysis restricted to regions showing increased activation during neurofeedback (as defined based on meta analytic coordinates from Emmert et al., 2016) revealed no significant differences between self‐regulation with neurofeedback compared to self‐regulation without neurofeedback (FDR corrected, *q* < 0.05). Deliberately applying a liberal correction threshold for decreasing the likelihood of false negatives in the our small sample revealed activation differences in the left anterior striatum, right aINS and left visual cortices and lower activation in the bilateral posterior striatum/thalamus remained, cluster corrected using Monte‐Carlo simulations with an initial threshold of *p* < 0.01.

## 5 DISCUSSION

### 5.1 Brain activation in response to neurofeedback during gradual self‐regulation

The main aim of this study was to identify activation related to neurofeedback processing during neurofeedback‐guided self‐regulation. We investigated this research question through analysis at whole‐brain level, in the striatum, a key region implicated in feedback and reward processing, and within a whole network of regions that reliably shows increased activation during neurofeedback as identified by a recent meta‐analysis.

We could replicate recent meta‐analytical findings (Emmert et al., 2016) within a single sample with regard to joint activation of cognitive control areas and areas involved in feedback learning by observing extended activation increases in prefrontal control hubs (pACC, lateral and posterior PFC) as well as regions involved in feedback and reward processing (aINS, striatum, visual cortices), the thalamus and deactivation in the default network across different mental tasks during neurofeedback. ROI analysis focussed on the striatum revealed significantly higher activation during gradual self‐regulation with rather than without feedback, suggesting that during neurofeedback, the observed striatum modulations reflect feedback learning and not self‐regulation per se, as participants achieved successful self‐regulation already without receiving feedback (for an extensive discussion of self‐regulation in the sample see Sorger et al., 2018) and both self‐regulation conditions did not differ with regard to the provided visual markers of task performance, that is absolute distance to target level. As participants were engaged in different self‐regulation task domains, the observed increase in activation was not related to a specific task domain, but specifically driven by neurofeedback. Subsequent analysis on the relationship between visual information provided during neurofeedback and striatum activation showed that more accurate neurofeedback performance was accompanied by an increased BOLD‐signal level in the anterior striatum in a late time window (8–14 s after a particular neurofeedback value was visually displayed), suggesting that the observed striatal activation increases during neurofeedback are indeed reflecting the processing of feedback information. While ROI analysis revealed increased activation during neurofeedback compared to self‐regulation without neurofeedback in the anterior striatum, we failed to detect activation differences during voxel‐wise analysis within the network of regions commonly involved in neurofeedback (Emmert et al., 2016). As we cannot exclude the absence of activation differences within other regions of the network (especially in feedback processing regions and visual areas as suggested by liberal cluster corrected analysis), further research with higher statistical power is needed to describe the distribution of activation within the whole network in comparison to self‐regulation without neurofeedback, as the sample size of the given study constituted a limitation with regard to statistical power, as well as a potentially slight variance introduced by different MR systems.

Overall, our findings are in line with recent theoretical approaches that suggest different sub‐components of the neurofeedback network for feedback processing and self‐regulation (Sitaram et al., 2017). While the anterior striatum appears to serve a unique function in response to neurofeedback, especially the lateral PFC and the ACC (of the network activated during neurofeedback in this study) have been defined as key regions in cognitive control in general (MacDonald, Cohen, Stenger, & Carter, 2000). Both regions are also jointly activated during various task modalities that involve cognitive control, including emotion regulation (Etkin, Egner, & Kalisch, 2011; Goldin, McRae, Ramel, & Gross, 2008), response inhibition (Cai et al., 2015) and attentional control (Weissman, Gopalakrishnan, Hazlett, & Woldorff, 2004), supporting their role as the general basis of self‐regulation. Fittingly, participants were instructed to dynamically engage and disengage from their mental task during self‐regulation (indeed participants confirmed to have followed these general strategies closely, see Sorger et al., 2018), thereby shifting their focus of attention to and away from the mental content driving the feedback, modulating activation in their attentional system during self‐regulation as in accordance with the observed prefrontal and parietal activation.

To our knowledge, this is the first study providing evidence for a linear relationship between the provided visual neurofeedback information and activation increases within the striatum. Conforming to the observed modulations of striatum activation by neurofeedback, most theoretical approaches on neurofeedback underline the importance of reward processing during neurofeedback, although several different working mechanisms have been proposed (for a recent overview see Sitaram et al., 2017). As the parametric activation paradigm applied in this study differs from previous research regarding the possibility to receive rewarding neurofeedback by up‐ as well as by down‐regulating activation adequately in relation to the target level, it remains open whether graded neurofeedback, as employed here, recruits the striatum differently compared to neurofeedback studies aiming at maximizing the neurofeedback magnitude, as task load and reward probability are known to modulate activation strength and timing of reward system activation (Cardinal, 2006; Stalnaker, Calhoon, Ogawa, Roesch, & Schoenbaum, 2012). Additionally, it would be important for further research to examine strategically whether modulating the reward contained in neurofeedback can be used to optimize its influence on the striatum, for example by investigating the effect of monetary reward for performance. Furthermore, to ensure that participants could optimally perform gradual self‐regulation in both conditions, and focus on the relevant marker of performance (i.e. either derived from neurofeedback or in the no‐feedback condition from introspection), no blinding was applied in the current study. As the lack of (double) blinding constitutes a limitation of our design, future research should investigate the effects of blinding on the reward system during self‐regulation with neurofeedback.

### 5.2 The influence of neurofeedback on different stages of reward processing

We also investigated how neurofeedback influences different stages of reward processing. In the current study, reward values were assigned to the distance between the instructed target activation level and the achieved activation level, which was updated every 2 s. Interestingly, the observed neurofeedback effect was substantially delayed (a significant difference between both training conditions was observed only in a time window 8–14 s after a corresponding feedback information was provided). Taking into account that rtfMRI‐based neurofeedback is delayed over several seconds, the reward information provided during and shortly after a mental action is unrelated to the neural activity subserving the mental action itself. It remains to be determined if reception of conflicting reward information during performance of a mental action leads to alterations in neurofeedback processing, as besides attributing value to a certain stimulus, the striatum also detects relations between performed actions and rewards (FitzGerald, Schwartenbeck, & Dolan, 2014; Haruno et al., 2004; Kim, Sul, Huh, Lee, & Jung, 2009) and predicts when a reward should occur (Kohrs et al., 2012). Additionally to receiving noncorresponding feedback during or shortly after a mental action, the predictability of reward information is also reduced due to noise or other confounding factors that distort the neurofeedback signal. Violations of reward expectancies as well as uncertainty of receiving rewards lead to alterations in striatum activation (Kohrs et al., 2012; McClure et al., 2003; Pagnoni et al., 2002). Both uncertainty and conflicting reward information could contribute to the difficulty to detect an immediate neurofeedback response in an early time window after the feedback is presented. Focusing on creating more direct closed‐loop approaches (El Hady, 2016; Potter, El Hady, & Fetz, 2014), for example, by neurofeedback‐guided brain stimulation systems that stimulate the striatum directly, could help to detect optimal time windows for operant conditioning and increase the efficacy of neurofeedback strongly.

With respect to the interpretability of the current results regarding different phases of reward processing, it is also to be noted that different stages of feedback processing do not only differ in time, but also recruit different sub‐regions of the striatum (Balleine et al., 2007; Sleezer & Hayden, 2016; Tanaka et al., 2004). As the current study aimed at describing the temporal relationship between visual neurofeedback information and striatum activation for the first time, regions of interest were chosen based on meta‐analytic peak coordinates on reward processing in fMRI, to increase the probability of detecting reward related activation. Due to this region‐of‐interest selection approach, different anatomical sub‐regions of the anterior striatum contribute to the observed activation, and as a consequence we cannot make any strong claims regarding the exact anatomical sub‐structures underlying this activation pattern.

However, the ventral section of putamen and caudate indeed have been shown to create reward predictions using temporal information (Hiebert et al., 2014) supporting the interpretation that here temporal properties of reward information are crucial. Especially anterior caudate has also been associated with biasing actions based on reward information (O'Doherty et al., 2004; Tanaka, Balleine, & O'Doherty, 2008; Tricomi, Delgado, & Fiez, 2004; Valentin, Dickinson, & O'Doherty, 2007), suggesting that the ROI signal represents merged processes of reward prediction and action selection, thereby reflecting the interwoven transitions between different reward processing stages in the striatum (Haber et al., 2000).

### 5.3 Linking mental actions to the information contained in neurofeedback

Neurofeedback differs from other domains of feedback learning during the learning process in that actions driving the reward are purely mental actions. To understand which mental actions lead to rewards, participants rely on introspection. The conscious monitoring of mental actions requires meta‐awareness, which recruits a distributed network of areas, including the pACC and the insula (Schooler et al., 2011). In a loop‐like fashion, these hubs have been suggested to interact with other higher‐order cognitive networks involved in working memory and attention, the striatum, the thalamus and regions recruited by the specific self‐regulation task during neurofeedback (Emmert et al., 2017; McCaig, Dixon, Keramatian, Liu, & Christoff, 2011; Sitaram et al., 2017). Neural‐feedback loops between these networks and the striatum could be crucial in identifying a relationship between mental actions and corresponding reward values. The complex pattern of continuous top‐down input from other regions to the striatum during reward processing (Haruno & Kawato, 2006), could be an important contributing factor for the observed delay in striatum reactivity to neurofeedback.

However, action‐effect mapping is not selectively dependent on conscious introspection (Hommel, 1996). Accordingly, recent studies (Ramot, Grossman, Friedman, & Malach, 2016) demonstrated that covert neurofeedback, that is, during which participants are not aware of the fact that they received neurofeedback, was accompanied by increased striatum activation (Ramot et al., 2016). Both automatic reward processing as well as conscious self‐regulation have been argued to be crucial in neurofeedback (Sitaram et al., 2017) but a mechanistic model of how automatic and subsequent stages of reward processing interact during neurofeedback is still lacking. For future research to tackle this issue, the temporal properties of neurofeedback should be taken into account because action‐reward mapping is known to be strongly influenced by the delay of a reward (Dobryakova & Tricomi, 2013; Tanaka et al., 2004).

## 6 CONCLUSION

This study demonstrates that neurofeedback contributes to self‐regulation through regions involved in feedback and reward processing, which share activation between different mental tasks. Focussing on the striatum as a key region in reward processing, we demonstrated increased activation in the anterior striatum during self‐regulation with neurofeedback, which correlated with self‐regulation success. The substantial delay in the observed effect suggests that these modulations reflect later stages of reward processing beyond simple detection of external rewards, but further research is needed to understand the mechanisms of neurofeedback reward learning. As trained associations between actions and rewards (for example during operant conditioning) are key to learning, the given results provide a promising outlook for neurofeedback to facilitate learning with the potential for operant conditioning of mental actions.

## ACKNOWLEDGMENTS

The authors would like to thank Florian Krause for valuable theoretical input.

## CONFLICT OF INTEREST

None declared.
